# Molecular characterization of divergent isolates of *Citrus bent leaf viroid* (CBLVd) from citrus cultivars of Punjab, Pakistan

**DOI:** 10.3389/fgene.2022.1104635

**Published:** 2023-01-12

**Authors:** Amjad Ali, Ummad ud Din Umar, Syed Atif Hasan Naqvi, Muhammad Taimoor Shakeel, Muhammad Nouman Tahir, Muhammad Fahad Khan, Muhammad Tanveer Altaf, Fatih Ölmez, Abdelfattah A. Dababat, Zia ul Haq, Muhammad Azhar Nadeem, Rüştü Hatipoğlu, Faheem Shehzad Baloch, Yong Suk Chung

**Affiliations:** ^1^ Department of Plant Pathology, Faculty of Agricultural Sciences and Technology, BahauddinZakariya University, Multan, Punjab, Pakistan; ^2^ Faculty of Agricultural Sciences and Technologies, Department of Plant Protection, Sivas University of Science and Technology, Sivas, Turkey; ^3^ Department of Plant Pathology, The Islamia University Bahawalpur, Bahawalpur, Punjab, Pakistan; ^4^ Department of Plant Protection, Faculty of Agricultural Sciences, Ghazi University, Dera GhaziKhan, Punjab, Pakistan; ^5^ International Maize and Wheat Improvement Centre (CIMMYT), Emek, Ankara, Turkey; ^6^ Department of Field Crops, Faculty of Agriculture, Kirsehir Ahi Evran Universitesi, Kirsehir, Turkey

**Keywords:** biological indexing, RT-PCR, sequencing analysis, phylogenetic analysis, citrus orchards

## Abstract

Citrus viroid infection is emerging as a serious threat because of its efficient systemic movement within the host plant and its quick spread due to contaminated pruning tools. A survey was conducted to investigate the primary distribution and molecular characterization of *Citrus bent leaf viroid* (CBLVd) and its variants in different citrus cultivars. A total of 154 symptomatic citrus samples were collected and detected by RT‒PCR with newly designed specific primers with the incidence of 36.33%. During biological indexing study on Etrog citron, expressions of reduced leaf size, yellowing with a light green pattern, and bending were observed. Amplified products were sequenced and analyzed using a nucleotide BLAST search, which showed 98% homology with other CBLVd isolates. The results of the phylogenetic tree analysis showed the presence of two main groups (A and B), with the predominant variants of CBLVd, i.e., CVd-I-LSS (Citrus viroid Low Sequence Similarity) sequences, clustering in subgroup A1 along with newly detected CVd-I-LSS from Palestinian sweet lime (Citrus limettioides), which has been identified as a new host of CVd-I-LSS in Pakistan. Further analysis of the sequences in subgroup A1 showed that the variant of CVd-I-LSS infecting citrus cultivars had a close relationship with isolates reported from China, Japan, and Iran, which may have resulted from the exchange of planting material. This study also unveiled the variability in nucleotide sequences of CBLVd, which made it unable to be detected by old primers. The results of this study indicate that the widespread presence of divergent variants of CBLVd is a major concern for the citrus industry in Pakistan and other countries where virulent isolates of CBLVd are prevalent. These findings suggest the need for future research on effective management and quarantine measures to stop the spread of CBLVd.

## 1 Introduction

Citrus, is known for its sourness, widely cultivated fruit crop around the world and belongs to *Rutaceae* family. Pakistan cultivates citrus on 206,569 hectares, producing 2.36 million tonnes. The Punjab Province, with its favorable environmental conditions, soil compatibility, and adequate water availability, has the highest citrus production in the country compared to other provinces (Memon and Kasbit, 2017; Mushtaq et al., 2022). Despite being grown in optimal conditions, the yield of citrus fruit falls short of its potential due to the presence of various pests and diseases, particularly viroids (Ali et al., 2022; Bakhtawar et al., 2022). Viroids are the smallest, unencapsidated, circular with ssRNA of 246 to 434 nucleotides and covalently closed plant pathogens (Chambers et al., 2022). Viroids are known to primarily infect citrus species, and eight different viroids have been identified within this host. These viroids belong to the family *Pospiviroidae* and are divided into four distinct genera (Mazhar et al., 2014; Cottilli et al., 2019; Iftikhar et al., 2019). Members of the *Pospiviroidae* family are characterized by their rod-like structures. These viroids have been found to replicate within the nucleus of host cells (Di Serio et al., 2014). Transmission of citrus viruses and viroids has been significantly assisted by both mechanical and vegetative methods. Citrus viruses have also been reported to be successfully transmitted through horticulture tools, during grafting, pruning and cutting (Fuji et al., 2022; Hadidi et al., 2022).

*Citrus bent leaf viroid* (CBLVd), also known as *Citrus viroid-*I (CVd-I), is a member of family *Pospiviroidae* and is included in the genus *Apscaviroid* (Ito et al., 2001; Chebli and Afechtal. 2018; Donovan et al., 2020). Pruning tools and grafting have been documented to transmit the CBLVd effectively in different citrus varieties regardless of rootstock. CBLVd is widely distributed in world citrus growing areas including United Arab Emirates (UAE), Italy, Israel, Pakistan, China, Cambodia, Australia, Malaysia, Iran, Japan, and recently in Argentina (Khoo et al., 2017; Iftikhar et al., 2019; Donovan et al., 2020; Palacios and Figueroa, 2022). Three variants of CBLVd have been identified to date, which have been found in different citrus cultivars. These variants, called CVd-Ia, CVd-Ib, and CVd-I-LSS, are distinguished from one another based on their genomic nucleotide sequences, which range in size from 327 to 329 nt, 315 to 319 nt, and 325 to 330 nt, respectively (Ito et al., 2000; Gandia and Duran-Vila, 2004; Chiaki and Ito, 2020; Zhou et al., 2020). A variant of CVd-I-LSS (Low sequence similarity) with 325–330 nucleotide bases, referred to as the Pakistan variant, has been reported. This variant exhibits a sequence identity of 83–85 percent with CBLVd. However, the sequence identity of this variant is lower compared to the other two variants, which show a higher sequence identity of 93–100 percent with CBLVd (Wu et al., 2014).

The CVd-I-LSS variant has been identified first time from Japan where indexing of this variant was conducted on Etrog citron (*Citrus medica* L.), which displayed severe epinasty symptoms (Ito et al., 2000). CVd-I-LSS differs from other variants due to variations in its rod-type secondary structures of the upper and lower strands, as well as in its central conserved region (CCR), pathogenic (P), right terminal (TR), and variable (V) domains. The central conserved region of Apple scar skin viroid (ASSVd), pathogenicity P, and left terminal T1 domain of Citrus exocortis viroid (CEVd) are included in the composition of CVd-Ib (Ashulin et al., 1991). Furthermore, the CVd-Ia variant of citrus viroid shared more than 90% sequence identity with other species of viroid, but some variants of CEVd had lower sequence similarities to other variants at less than 90% (Ben-Shaul et al., 1995).

The prevalence of CVd-1-LSS has been steadily increasing as it spreads from one location to another due to a lack of awareness about quarantine measures. The CVd-I-LSS strain was reported in sweet oranges, succri, sweet limes, red blood sweet limes, Kinnow mandarins, Akemi, and Nishirkaori in Pakistan (Wu et al., 2014). The symptoms that this variant causes on host plants include reduced plant growth, epinasty (a condition where the leaves bend downwards), yellowing of the leaves, mild necrosis (death) of the mid-vein of the leaves, and pitting (small indentations) on the bark (Semancik et al., 1997). Ito et al. (2000) reported that when the extracted RNA from CVd-I-infected plants was inoculated to a host plant, it caused moderate leaf bending and severe epinasty (a condition where the leaves bend downwards). Iftikhar et al. (2019) used the sap from plants infected with CVd-I to inoculate healthy host plants. After few months, these healthy host plants showed symptoms such as leaf bending, chlorosis, leaf rolling, and mild necrosis of the petiole. A mixed infection of CBLVd, Citrus Back Cracking Viroid (CBCVd), and Citrus Dwarfing Viroid (CDVd) can cause stem pitting, reduced plant growth, and bark cracking symptoms (Roistacher et al., 1993; Vernière et al., 2006). Despite the fact that, CBLVd has been found in several Asian countries, but there is a shortage of research on its characterization and its sap transmissibility. It is worth noting that citrus viroids have been shown to not only impact the citrus industry but also hinder per acre yield. The purpose of this study was to use RT-PCR to determine the prevalence of CBLVd variants and to characterize them in different citrus cultivars in Pakistan.

## 2 Material and methods

### 2.1 Collection of samples

154 citrus samples were collected from seven different areas in Punjab known for their citrus production (Sargodha, Toba Tek Singh, Sahiwal, Rahim Yar Khan, Multan, Layyah, and Khanewal). These samples were selected based on the presence of specific viroid symptoms such as stem pitting, bark cracking, gumming, stunted growth, and leaf curling. Samples were collected from different citrus cultivars viz., Kinnow and Feutrell Early “mandarin”, Sweet Lime, Tangerines, Grapefruit, Sweet orange, Lemon, and tangelos (Table 1). The majority of the samples were collected from old “Mandarin” Kinnow plants that displayed severe stem pitting, bark cracking, and gummosis. The infected leaves and bark were disinfected using a 10% sodium hypochlorite solution and stored in RNAfterTM (GeneMark Bio, Koerea) until the RNA was extracted.

**TABLE 1:** Collection of infected citrus samples from different locations.

Cultivar’s name	*Observed symptom	No. of samples	Locations
Kinnow “mandarin”	BC S&C, SG, LB, CG	53	LYH, TTS, RYK, MTN, KWL
Feutrell Early “mandarin”	PN, BC S&C, EPI	12	LYH, RYK, MTN
Sweet Orange	CG, SG, SP	32	LYH, RYK, MTN, KWL, SGD
Grapefruit	C.C, SP, SG	21	MTN, KWL, SGD, SWL
Sweet Lime	BC S&C, LB	21	KWL, LYH, SGD, SWL
Lemon	BC S&C	7	SGD, KWL, SWL
Tangerines	BC S&C, EPI, SP	8	SGD, SWL
Total		154	

BC S&B, Bark cracking of stem and branches; Epi, Epinasty; Stunting growth; YOL, Yellowing of old leaves; LB, Leaf bending; PN, Petiole necrosis.

### 2.2 RNA extraction of citrus samples

To extract total nucleic acid from citrus leaves, 100 mg of tissue from each sample was taken in an Eppendorf tube and ground with a sterilized plastic micro-pestle in liquid nitrogen. After grinding, 500 µl of TRIzol^®^ Reagent (Thermo Fisher Scientific, Invitrogen, United States) was added to the tube and vortexed vigorously. The tubes were then incubated at room temperature for a few minutes in a horizontal position. Following incubation, the tubes were centrifuged for 5 min at 12,000 rpm and the supernatant was transferred to new 1.5 ml microcentrifuge tubes. To clarify the supernatant, 100 µl of 5 M NaCl and 300 µl of chloroform were added to the sample tubes and mixed thoroughly. The samples were then centrifuged for 10 min at 12,000 rpm, and the top separated phase was transferred to a new 1.5 ml tube. Isopropanol was added in an equal volume and the tubes were incubated for 20 min at room temperature. After incubation, the tubes were centrifuged at top speed for 2 min, and the RNA was precipitated into a pellet. The RNA pellet was washed with 70% ethanol twice and dissolved in nuclease-free water. The concentration and quality of the extracted RNA was determined using a Nano-drop (Thermo scientific, Inc. United States) and the RNA was preserved at −70°C.

### 2.3 Synthesis of complementary DNA (cDNA)

Complementary DNA (cDNA) was synthesized according to the method of Ito et al. (2002) where 1 ml of 10 μM reverse primer of CBLVd (AR1 5′-CAG​GAA​CCA​CAA​GAA​GTC​T-3′) was used for preparation of cDNA with 3 μl of template (RNA), 1 μl of 10 M dNTPs and 7 μl of nuclease-free water. The mixture tube was incubated at 65°C for 5 min and immediately placed on ice for 2 min. The RT mixture was prepared by adding 5 μl of (5×) reaction buffer, 1 μl of M-MLV-RT, 1 μl of RNase Inhibitor and 1 μl of 25 mM MgCl_2,_ and finally, the reaction mixture was incubated in a Mycycler (Bio-Rad, United States) at 60°C for 60 min and 70°C for 15 min cDNA was synthesized and preserved at −20°C for future usage.

### 2.4 RT-PCR amplification

In this experiment, RT-PCR was used to detect CBLVd using newly designed primers called AF1-TCCTGTGGTGACACCCCTC and AR1-CAGGAACCACAAGAAGTCT. The PCR mixture was made up of a PCR master mix, the forward and reverse primers, cDNA, and nuclease-free water, total volume of 25 μl. The PCR profile included a denaturation step at 94°C for 5 min, followed by 30 cycles of denaturation at 94°C for 30 s, annealing at 54°C for 30 s, and extension at 68°C for 45 s. The final extension step was performed at 68°C for 7 min. The amplified PCR products were then separated by electrophoresis on a 1% agarose gel with ethidium bromide in 0.5 x TBE buffer. The gel was run at 90 V for 60 min and visualized under UV light using a Gel Documentation apparatus (Bio-Rad, United States).

### 2.5 Biological indexing of CBLVd

The recommended technique of Barbosa et al. (2005) and Roistacher (1991) was followed during the study for the confirmation of CBLVd under controlled conditions. The infected budwood of grapefruit and sweet lime with characteristic symptoms of petiole necrosis, yellowing and leaf bending were collected from district Sahiwal. Infected budwood was grafted on a 1.5-year-old Arizona 861-S1 Etrog citron plant with T-grafting method. The grafted plants were placed in controlled conditions with a temperature range of 25°C–28°C at night and 25°C–32°C in daytime. The symptoms were observed after 5–6 months on grafted plants.

### 2.6 Purification of PCR products and cloning

PCR products of CBLVd were purified using a PCR Purification Mini Kit from FAVORGEN (BioTek, United States) according to the manufacturer’s protocol. These purified products were then inserted into a vector (pCR2.1-TOPO, Invitrogen) for cloning. The transformation process involved placing the samples in a water bath at 42°C for 25–40 s, followed by transferring them to an icebox for 5 min. The samples were then placed in a LB medium containing ampicillin (100 mg/ml) and incubated in an orbital shaker at 37°C for 70 min at 200 rpm. To confirm the presence of recombinant cells, X-gal was applied to an LBA plate, which was then sealed with aluminum foil and incubated at 37°C for 45 min. Afterward, samples of the desired size were selected and sent for sequencing.

### 2.7 Sequencing and phylogenetic analysis

The cloned products were sequenced by Sanger technology in a single forward direction with a forward primer. The obtained sequences were cleaned and subjected to BLASTn on the NCBI site for their confirmation. The maximum similar sequence was copied and inserted in MEGA X software for the phylogenetic analysis and for alignment to check nucleotide variations of our obtained sequence with a previously reported sequence of CBLVd and variant CVd-I-LSS (Tamura et al., 2011).

## 3 Results

### 3.1 Observation of field symptoms

**FIGURE 1:**
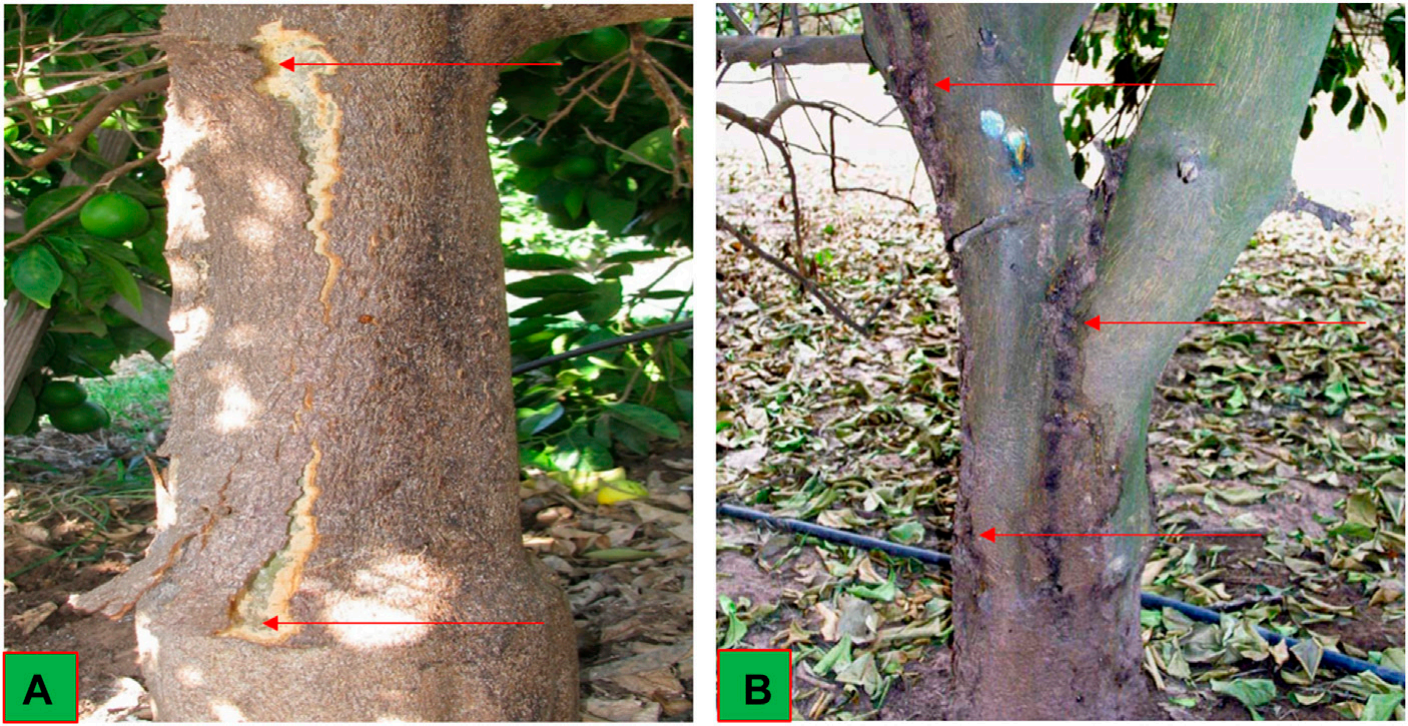
Infected citrus plants **(A)** Kinnow “mandarin” plant showing severe bark cracking and **(B)** Sweet lime plant showing stem pitting and gumming.

During the survey, it was observed that the citrus cultivars showed the typical symptoms of CBLVd, including stem pitting, bark cracking, gumming on stems and branches, petiole necrosis and yellowing of leaves. Bark cracking was found in the severe form on old Kinnow “mandarin” and sweet lime plants (Figure 1).

### 3.2 Detection of *citrus bent leaf viroid* (CBLVd) through RT-PCR

**FIGURE 2:**
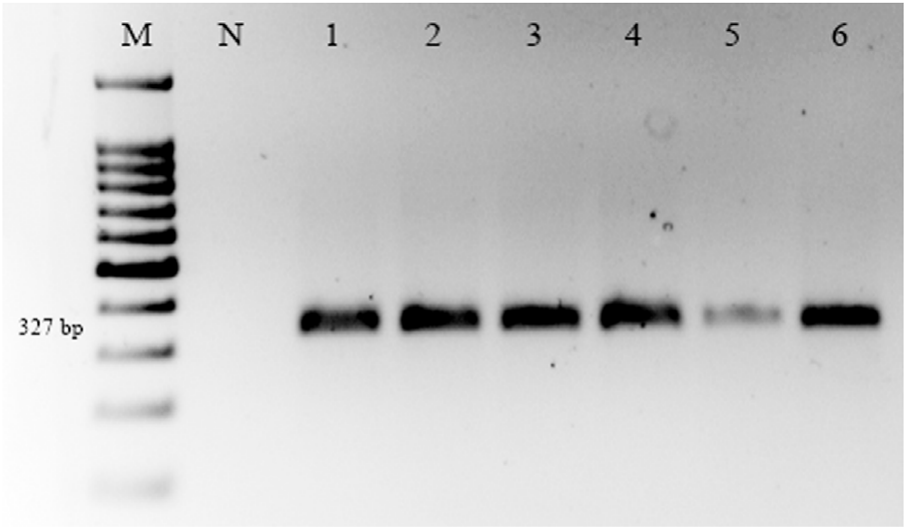
Amplification of CBLVd with 327 bp on a 1% agarose gel of samples collected from KWN, SWL, and SGD districts. Lane M is a 100 bp marker; Lane N is the negative control, and Lanes 1-6 are positive samples of CBLVd.

RT-PCR was used to detect CBLVd in 154 samples, and the results showed that 56 (36.36%) of the samples tested positive for CBLVd when using the newly designed specific primers. CBLVd was detected in samples from five out of six citrus varieties, including 24 samples from *C. reticulata*, 11 from *C. sinensis*, 10 from *C. paradisi*, 9 from *C. limetta*, and 2 from *C. tangerina* (Table 2) but it was not detected in *C. limon*. The positive samples showed an amplification of approximately 327 bp when analyzed using 1% agarose gel electrophoresis (Figure 2).

**TABLE 2:** Detection of CBLVd from collected samples by using RT-PCR.

Number of citrus cultivars detected withCBLVd (n)			Total				
1.1.1 District (N)	*C.*	*C.*	*C.*	*C.*	*C.*	*C.*	
	*reticulata*	*sinensis*	*paradisi*	*limetta*	*limon*	*tangerina*1	(+ve)
Layyah (35)	7 (19)	4 (12)	-	2 (4)	-	-	13
Toba Tek Singh (07)	4 (7)	-	-	-	-	-	4
Rahim Yar Khan (22)	5 (13)	3 (9)	-	-	-	-	8
Multan (25)	4 (15)	2 (6)	3 (7)	-	-	-	9
Khanewal (28)	4 (11)	1 (3)	2 (4)	3 (7)	0 (3)	-	10
Sargodha (23)	-	1 (2)	3 (7)	3 (7)	0 (2)	1 (5)	8
Sahiwal (11)	-	-	2 (3)	1 (3)	0 (2)	1 (3)	4
Total (154)	24 (65)	11 (32)	10 (21)	9 (21)	0 (7)	2 (8)	56

(N) Total number of samples.

(n) Total number of cultivars.

(-) absence of characteristic symptoms of viroids in the field.

**FIGURE 3:**
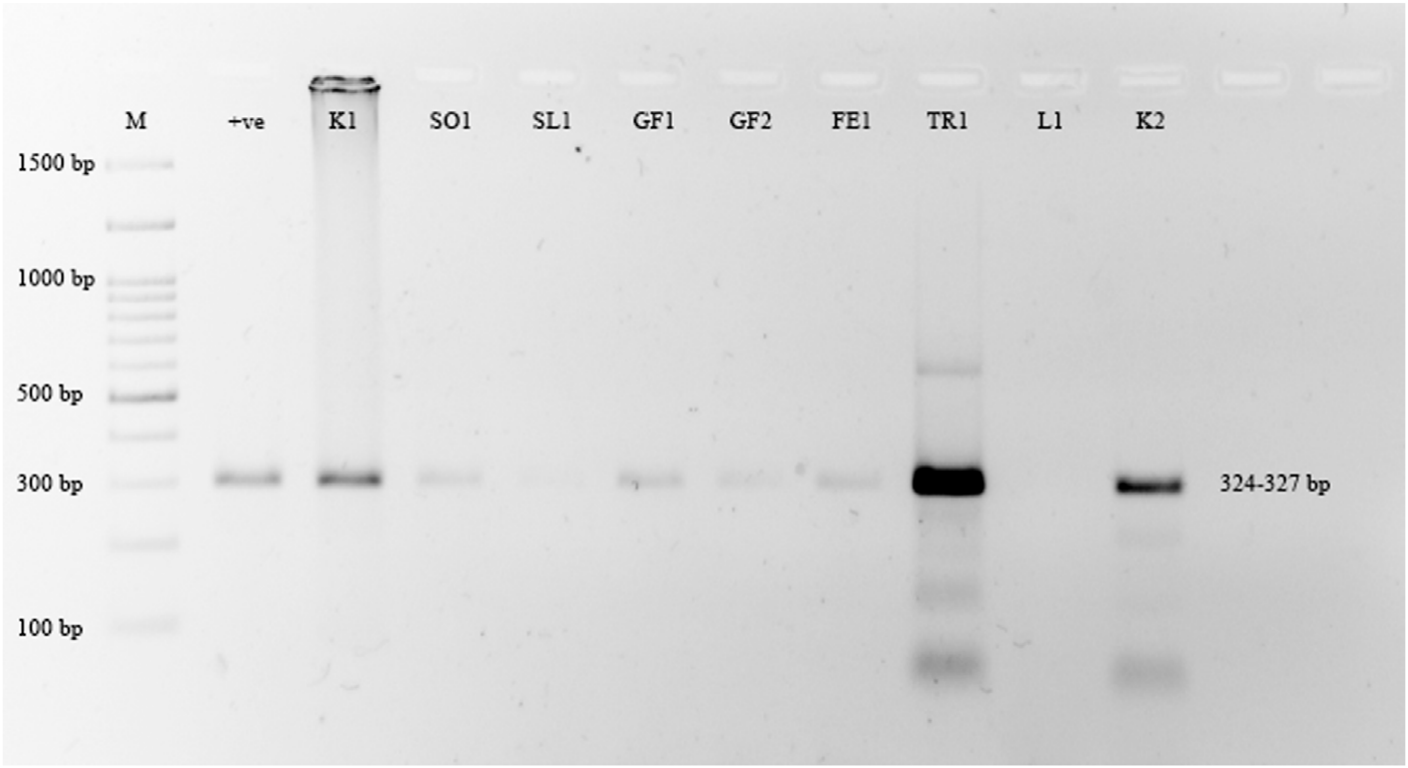
Amplification of CBLVd variant CVd-I-LSS with 324–327 bp from samples collected from all districts. Lane M is 100 bp marker; Lane 1 is positive control and Lane 2 (Kinnow “mandarin”); 3 (Sweet orange), 4 (Sweet lime), 5 (Grapefruit), 6 (Grapefruit), 7 (Feutrell Early “mandarin”), 8 (Tangerine), 9 no amplification from lemon sample and lane 10 is positive for Kinnow “mandarin”.

Grapefruit (*C. paradisi*) samples from the Multan, Khanewal, and Sargodha districts had the highest incidence of CBLVd at 47.61%, compared to other cultivars. *C. limetta* had an incidence of 42.85%, *C. reticulata* 37.5%, *C. sinensis* 34.37%, *C. tangerina* 25%, and *C. limon* did not show any signs of infection. Lemon (*C. limon*) samples collected from Khanewal, Sargodha, and Sahiwal were tested for CBLVd using the newly designed primers and did not yield any positive results (Figure 3). Sweet lime (*C. limetta*) samples collected from Khanewal, Sargodha, and Sahiwal had an incidence of 42.87% for CBLVd. The variant CVd-I-LSS of CBLVd was identified in Palestinian sweet lime (*Citrus limettioides*), which expands the known host range of CBLVd in Pakistan. The incidence of CBLVd by cultivar is shown in Table 3.

**TABLE 3:** Cultivars-wise, incidence of CBLVd in tested samples through RT-PCR.

Citrus cultivars	Tested samples	Positive sample for CBLVd	Incidence percentage (%)
*C. reticulata*	65	24	37.5
*C. sinensis*	32	11	34.37
*C. paradisi*	21	10	47.61
*C. limetta*	21	09	42.85
*C. limon*	07	00	00
*C. tangerina*	08	02	25
Total samples	154	56	36.36

### 3.3 Biological indexing

**FIGURE 4:**
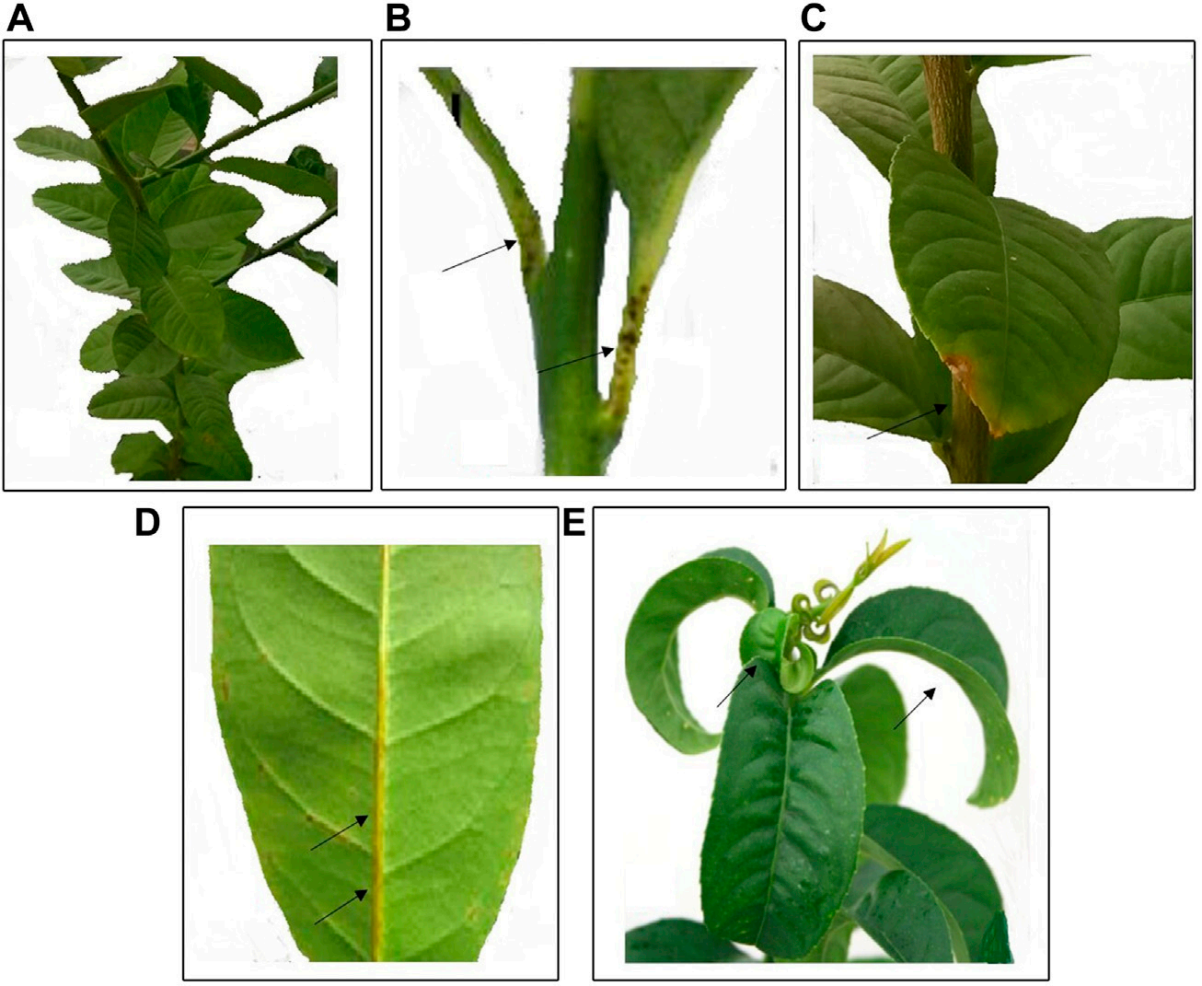
Biological indexing, **(A)** Control Etrog citron plant, **(B)** Petiole necrosis, **(C)** Leaf tip necrosis **(D)** Midrib necrosis with yellow color **(E)** Epinasty and moderate to severe leaf curling.

**FIGURE 5:**
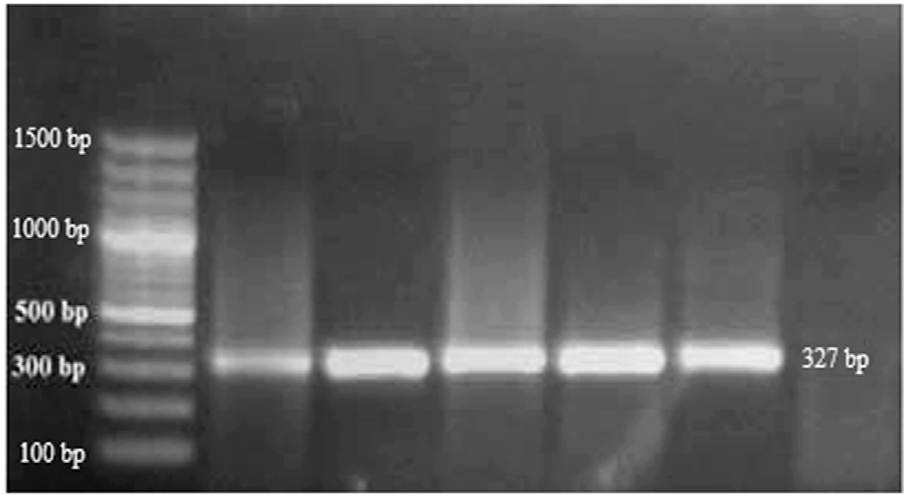
RT-PCR showed the positive results and presence of grafted samples of 327 bp on a 1% agarose gel.

Arizona 861-S1 citron plants grafted onto rootstock showed symptoms of moderate to severe leaf epinasty after 4–6 months under controlled conditions at a temperature of 28°C ± 4°C. Other symptoms such as leaf curling, petiole, midrib, and leaf tip necrosis with yellowing of leaves were also observed (Figure 4). RT-PCR was performed on the plants used for indexing, using specific primers designed for CBLVd. The PCR products, which had a size of 327 bp, were resolved on a 1% agarose gel (Figure 5).

### 3.4 Sequencing and phylogenetic analysis

BLASTn analysis showed that the sequences of the cloned RT‒PCR products had 100% similarity with CBLVd. BLAST results of isolate P1-236 (MN885661) collected from the Sahiwal district showed that it was a variant of CVd-I-LSS of CBLVd with 327 nt. The CVd-I-LSS variant showed 94% nucleotide identity with isolates from other parts of world. NCBI BLAST results of other two isolates P2-237 (MN885662) and P3-238 (MN885663) were collected from district Sahiwal showed 100% nucleotide identity with CBLVd. All obtained sequences of CBLVd along with other isolates are given in Table 4 with their country name, accession number and cultivar name.

**TABLE 4:** List of accession numbers of CBLVd obtained during the study and other previously deposited NCBI sites that showed maximum similarity with our isolates.

Isolate	Citrus cultivar	Geographic location	Accession no.	Organism	Reference
P1-236	*Citrus limettioides*	Sahiwal, Punjab	MN885661	CVd-I-LSS	This work
P2-237	*Citrus limettioides*	Sahiwal, Punjab	MN885662	CBLVd	This work
P3-238	*Citrus limettioides*	Sahiwal, Punjab	MN885663	CBLVd	This work
RB1	*Citrus sinensis*	Pakistan	KF726095	CVd-I-LSS	Wu et al. (2014)
SU4	*Citrus sinensis*	Pakistan	KF726096	CVd-I-LSS	Wu et al. (2014)
SL4	*Citrus limettioides*	China	KF726092	CVd-I-LSS	Wu et al. (2014)
SL1	*Citrus limettioides*	China	KF726091	CVd-I-LSS	Wu et al. (2014)
WS2	*Citrus* sp.	Japan	AB054640	CVd-I-LSS	Ito et al. (2001)
9 KS	*Citrus* sp.	Japan	AB054641	CVd-I-LSS	Ito et al. (2001)
NRCVO3	*Citrus sinensis*	South Africa	KT725631	CVd-I-LSS	Al Rwahnih et al. (2018)
SO1	*Citrus sinensis*	Pakistan	KF726098	CVd-I-LSS	Wu et al. (2014)
CB25	Volkamer lemon rootstock	Italy	MF421259	CBLVd	Rizzo et al. (2017)
A33	*Citrus sinensis* cv. Mosambi	Pakistan	FJ773263	CBLVd	Cao et al. (2009)
lot2	*Citrus* sp.	Thailand	KM214207	CBLVd	Tangkanchanapas et al. (2005)
CB20	Volkamer lemon rootstock	Morocco	MF421258	CBLVd	Chebli and Afechtal, (2018)
CBLVd-GH-Cl5	*Citrus clementina*hort. ex Tanaka	Morocco	MH200819	CBLVd	Chebli and Afechtal, (2018)

**FIGURE 6:**
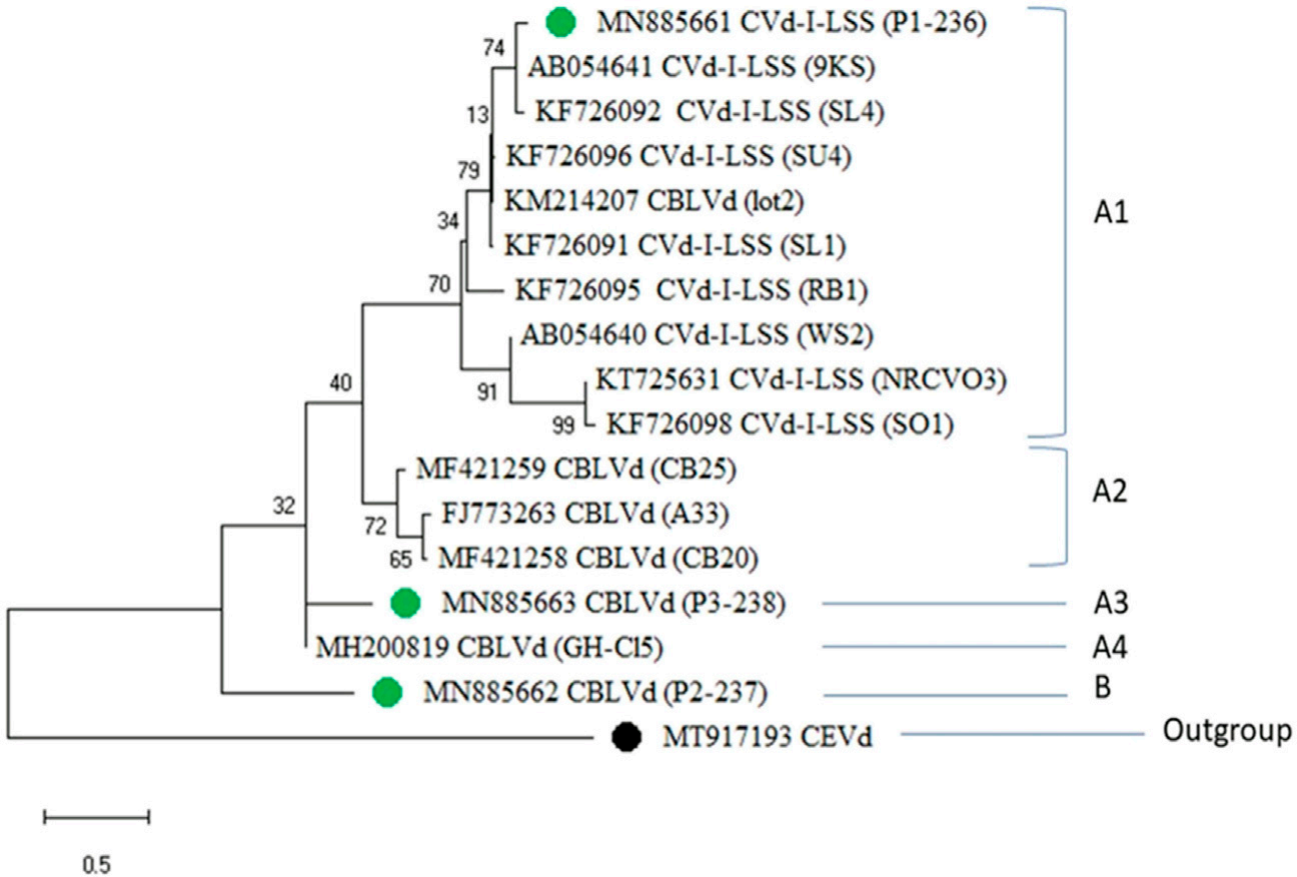
Phylogenetic tree of CVd-I-LSS and CBLVd sequences obtained during the current study (green spots) with an out-group of CEVd (black spot) showing the similarities with reported sequences from other countries.

The maximum likelihood tree depicted that the CBLVd isolates detected in Pakistan were closely related to isolates previously reported from Pakistan (KF726092, KF726091, KF726096, KF726095, and KF726098), with a nucleotide identity of 100%, and distantly related to isolates from Japan (AB054641) and South Africa (KT725631). The tree generally showed two major groups, A and B (Figure 6). Group A was further divided into subgroups A1, A2, A3, and A4. CEVd (accession number MT917193) was used as an outgroup to root the phylogenetic tree.

Group A1 included ten CVd-I-LSS variants, nine of which were retrieved from NCBI and one of which was a newly characterized isolate from Pakistan (MN885661, marked in green). The variants in Group A1 showed 94.36% sequence identity with sequences reported from Thailand (KM214213), China (KF726093), Thailand (KM214209), and Japan (AB019509). The sequences reported from Pakistan (KF726094) showed 100% and 98.33% identity with the sequence (AB054641) from Japan. Some other isolates, reported from Iran and Pakistan, showed low sequence similarity of 88%–89%. Subgroup A3 was made up of a single isolate (MN885663) characterized from Pakistan. Another isolate from Pakistan (MN885662) was relatively more divergent and fell into a separate clade, called “B.”

## 4 Discussion

Samples of citrus plants infected with viroids were collected from seven districts in Punjab, Pakistan, including Sargodha, Toba Tek Singh, Sahiwal, Layyah, Multan, Khanewal, and Rahim Yar Khan, in order to determine the natural host range of viroids in the region during 2018-2019. These samples were collected from different citrus cultivars exhibiting symptoms such as leaf bending, epinasty, petiole necrosis, bark cracking, stem pitting, and severe stunting. A total of 154 collected samples were tested for the presence of CBLVd viroid using RT-PCR. The protocol for RT-PCR was optimized for the identification of CBLVd.

After several attempts at using specific reported primers (Wang et al., 2009) for the detection of CBLVd through RT‒PCR were unsuccessful due to a lack of amplification. To optimize the RT‒PCR procedure, the sequences of the reported primers were analyzed using BLAST, and it was found that they had no significant sequence homology with CBLVd. This was further confirmed by aligning the sequences of CBLVd in the database with the primers, which did not match many of the sequences of CBLVd (Supplementary Figure S1). As a result, new back-to-back primers (CBLVd-AF1/CBLVd-AR1) were designed from several sequences retrieved from NCBI data. All samples were then tested using the new primers, which produced highly specific CBLVd results. Molecular characterization confirmed that Palestinian sweet lime was identified as a new host for CBLVd for the first time in Pakistan during the study.

The CVd-I-LSS variant was first reported to infect “Shiranui”, a citrus cultivar and sweet orange plant in Japan (Ito et al., 2001). Later, Satsuma mandarin (*Citrus unshiu*) was also reported as a new host of CVd-I-LSS from Iran by Alavi et al. (2005). In Pakistan, this CVd-I-LSS variant was reported only on *C. sinensis*, *C. limettioides* “Succri” sweet orange, “Kinnow” mandarin (*C. reticulata*) and “Red blood” sweet orange from Pakistan (Wu et al., 2014), and two citrus cultivars named Nishirkaori and Akemi imported from China were known to be infected by CVd-I-LSS. It can be speculated that new variant might have been introduced in Pakistan by nurseries traded through uncontrolled quarantine measures.

During the investigation, it was also found that sweet orange samples collected from Layyah district were infected with both CBLVd and CVd-V, while Kinnow “mandarin” had a mixed infection of CBLVd, CVd-III, CVd-IV, and CVd-V. In controlled conditions at 28°C–32°C, Arizona 861-S1 citron plants showed moderate to severe epinasty symptoms after 4–6 months in an indexing experiment. Other symptoms, including stunting, bark cracking, and yellowing of leaves, were also observed. Members of the genus Apscaviroid, which do not cause any specific disease in citrus plants, can induce symptoms of stunting, epinasty, and yield reduction in plants (Mughal, 2004; Cao et al., 2013). The synergistic effects of CBLVd, CVd-III, and CVd-IV can cause symptoms such as stem pitting, stunting, and bark cracking (Vernière et al., 2004; Vernière et al., 2006). Coinfection with CBLVd and CVd-V can induce symptoms such as changes in leaf shape and stunted plant growth (Serra et al., 2008). Ito et al. (2002) found that mixed infection of CBLVd with HSVd, CDVd, and CBCVd in controlled conditions induced Exocortis-like symptoms on the indicator citrus plant Etrog citron. Synergism among CBLVd, HSVd, and CDVd was significantly responsible for the decline in the canopy size of the trifoliate orange (*Poncirus trifoliate*) (Roistacher et al., 1993; Tamura and Nei, 1993). Trifoliata (*Poncirus trifoliata* (L.) Raf.) and citrange (*Citrus sinensis* x *P. trifoliata*) rootstocks have been found sensitive to infection by several viroids which exhibit different symptoms like bark scaling, dwarfing and yield reduction (Palacios and Figueroa, 2022). The increased use of these rootstocks has shown symptomatic evidences of viroids in the citrus growing countries. Recently, Bakhtawar et al. (2022) reported that the CBLVd infection in citrus cultivars effect the biochemical properties. There were less chlorophyll contents (Chlorophyll a, b and total Chlorophyll) observed in infected leaves as compared to healthy whereas total soluble phenolic (TSP), polyphenol oxidase (PPO) and phenylalanine ammonia lyase (PAL) activities were found higher in the CBLVd infected samples of citrus cultivars.

The cloned sequence of CBLVd collected from the Sahiwal region and its accession number were used for the construction of the phylogenetic tree. Generally, the distribution of the phylogenetic tree shows two major groups, i.e., A and B are subdivided into A1, A2, A3, A4, B1 and B2 depending on the genetic similarities. Group A1 consisted of CVd-I-LSS variants. These isolates of CVd-I-LSS showed maximum identity to one of newly characterized isolates (MN885661) considering that none of the CVd-I-LSS isolates fell into any other clade of phylogenetic tree. On the other hand, it shows 94.36% sequence identity with sequences reported from Thailand (KM214213), China (KF726093), Thailand (KM214209) and Japan (AB019509). Previously, the sequence reported from Pakistan (KF726095) showed 98.33% identity with sequence (AB054641) of Japan. Some other isolates showed 88%–89% low sequence similarity reported from Iran and Pakistan. Another newly identified isolate (MN885663) showed its divergent nature, as it did not share its clade (A3) with any other isolate.

CBLVd and the variant CVd-I-LSS were detected from *C. limettioides* by RT-PCR with specific newly designed primers where CVd-1-LSS showed 94%–100% sequence similarity with CBLVD isolates, and it is considered a new variant of CBLVd (Ito et al., 2000); however, it is not considered a new species because the species demarcation threshold for viroids is 80%. The third group has some changes in their nucleotide and showed a separate group from all the collected sequences (Altschul et al., 1997; Arif et al., 2005).

CBLVd was detected from different citrus cultivars in Punjab through RT-PCR with newly designed specific full-length primers for CBLVd. CBLVd was detected in *C. reticulata, C. sinensis, C. paradisi, C. limetta* and *C. tangerina*. Cloning and sequencing revealed that the CVd-I-LSS variant showed 94% identity with CBLVd.

## 5 Conclusion

The prevalence of CBLVd in different regions is likely due to the spread of contaminated bud wood and the unregulated trade of plant materials. In Pakistan, many citrus nurseries are managed by non-technical personnel, and infected tools and bud wood are significant sources of viroid proliferation. The presence and distribution of viroids in citrus groves of Pakistan reinforces the need for accurate diagnostics to support the certification programms which needs serious legislation and awareness to prevent the spread of CBLVd and other viroids that pose significant threat to industry and consequently the economy.

## Data Availability

The original contributions presented in the study are included in the article/Supplementary Material, further inquiries can be directed to the corresponding authors.

## Abbreviations

CBLVd, Citrus bent leaf viroid; RT-PCR, Reverse transcription polymerase chain reaction; CVd-I-LSS, Low sequence similarity; CVd-I, Citrus viroid I; CCR, Central conserved region; ASSVd, Apple scar skin viroid; CEVd, Citrus exocortis viroid; CBCVd, Citrus back cracking viroid; CDVd, Citrus dwarfing viroid; nt, Nucleotide; NCBI, National center for biotechnology Information; BLAST, Basic local alignment search tool; CVd-V, Citrus viroid V; HSVd, Hop stunt viroid; NaOCl, Sodium hypochlorite; SOC, Super optimal broth with catabolite repression.
