# Supplementary material for: Molecular characterization of divergent isolates of Citrus bent leaf viroid (CBLVd) from citrus cultivars of Punjab, Pakistan
Source: Front Genet. 2023 Jan 12;13:1104635. doi: 10.3389/fgene.2022.1104635 (PMC9878587; doi:10.3389/fgene.2022.1104635)
Supplement: Supplementary file 1 [file Table1.DOCX]

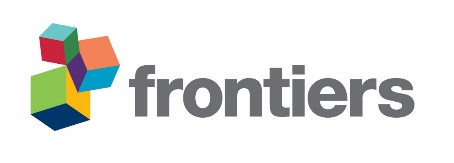
Supplementary Material (Figures)


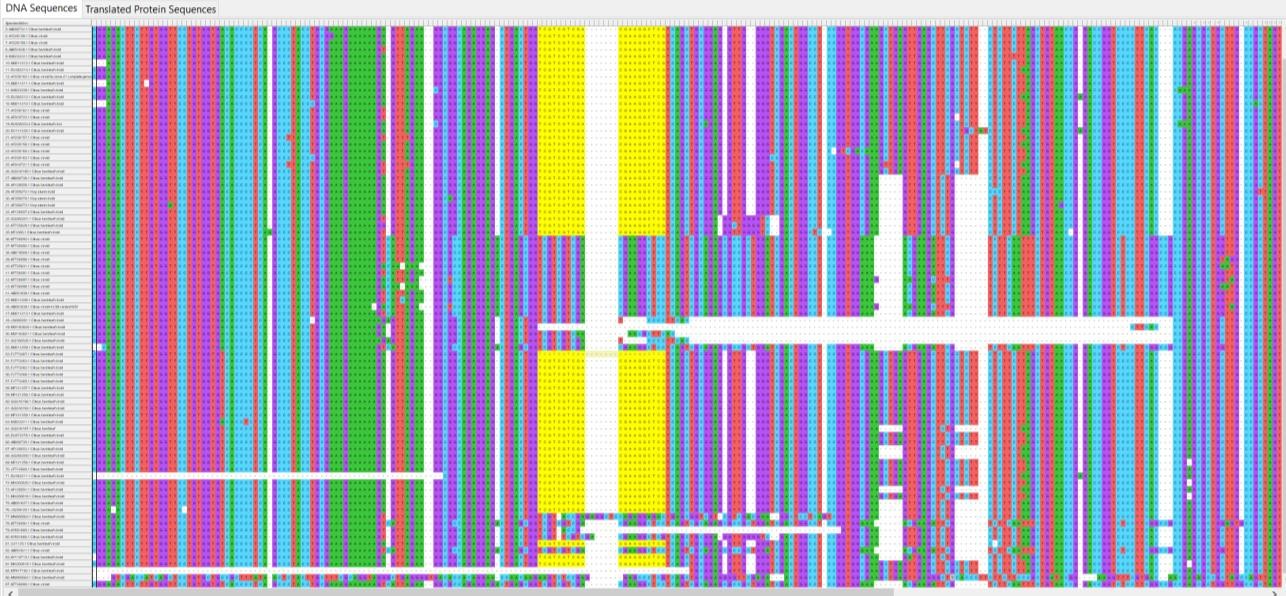


Figure 1. CBLVd Forword Old Primer.


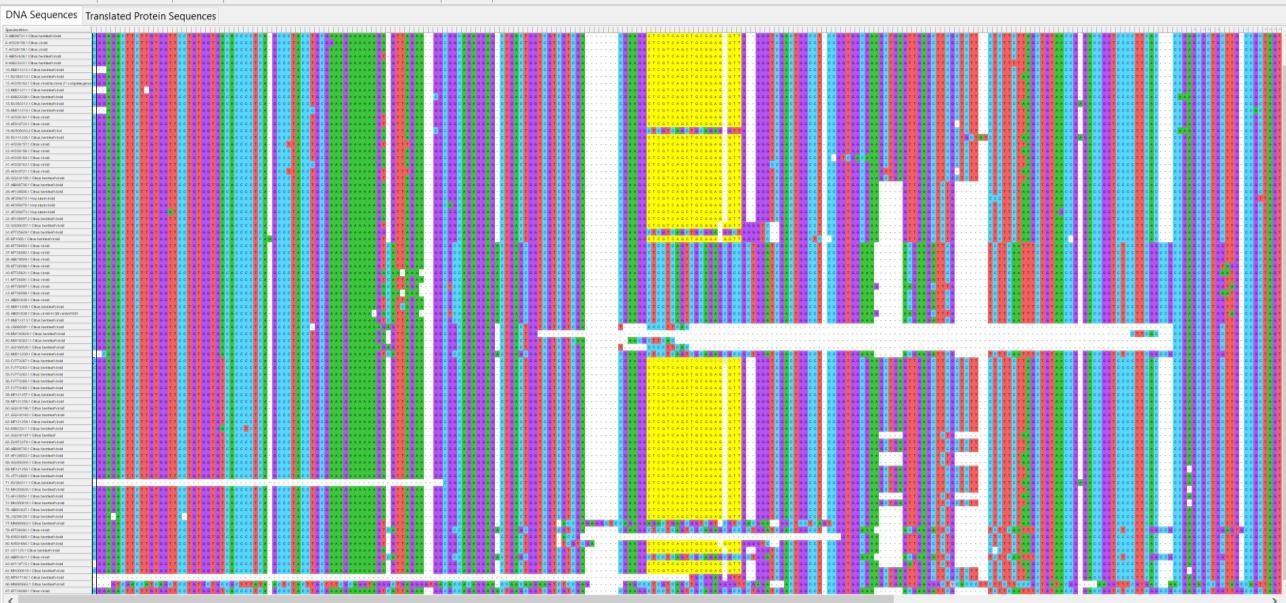


Figure 2. CBLVd Reverse Old Primer.


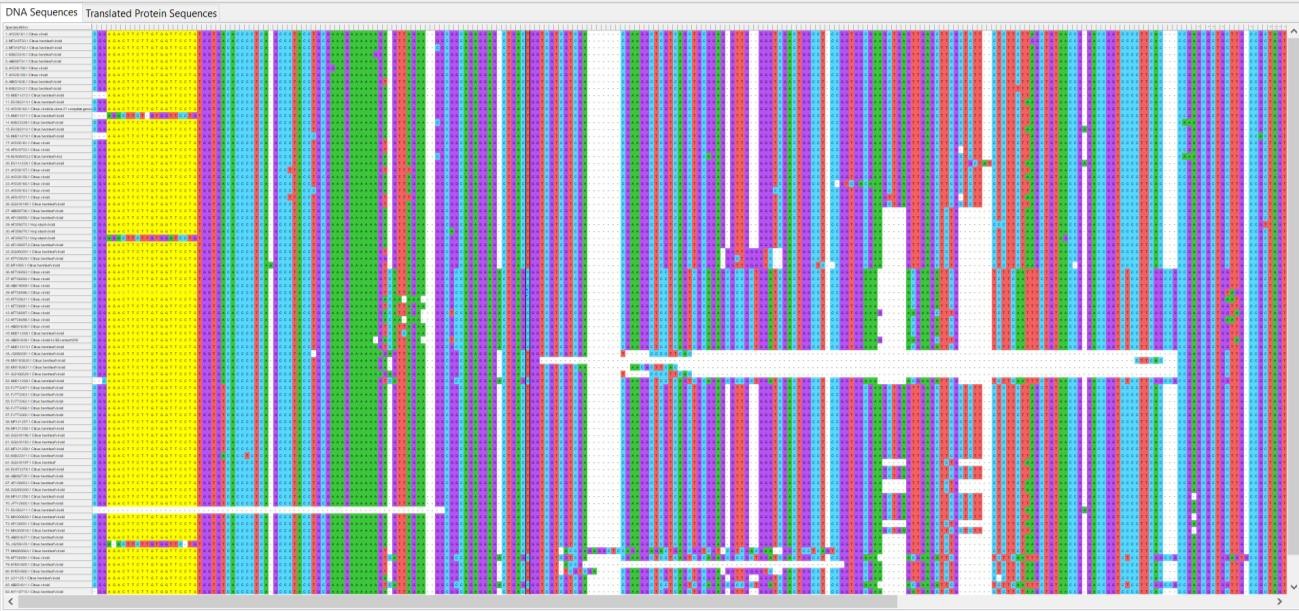


Figure 3. CBLVd AF1 New Primer.


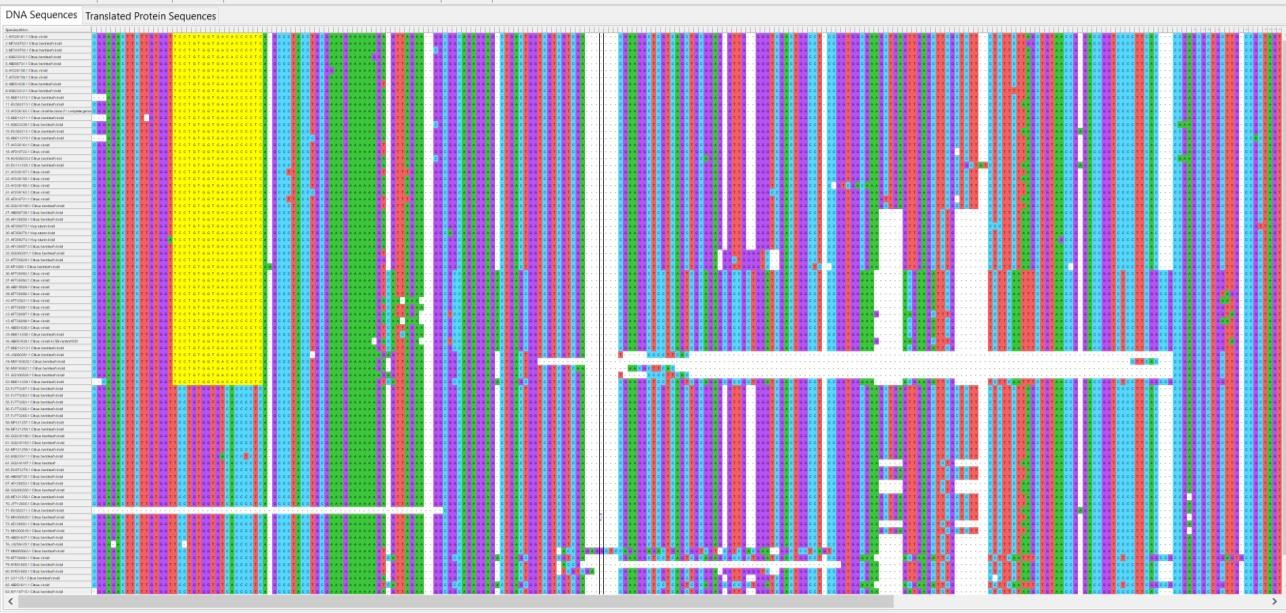


Figure 4. CBLVd AR1 New Primer.

**Figures Legends**

**Supplementary Figure 1.** Comparison of old and new primers sequence (Fıgure 1, 2, 3, and 4). The reported primers are not suıtable due to change or variation in CBLVd new emerging and old reported isolates. The alignment of the old and new primers sequences clearly showed the variation in Pakistani isolates.
